# A comprehensive historical and geolocalized database of mining activities in Canada

**DOI:** 10.1038/s41597-024-03116-3

**Published:** 2024-03-21

**Authors:** Clara Dallaire-Fortier

**Affiliations:** Department of Economic History, Lund University, Lund, Sweden

**Keywords:** Environmental economics, Economics, History, Industry, Geography

## Abstract

This paper introduces the MinCan database that presents mine-level estimates for the Canadian mining industry with a persistent annual coverage between 1950 and 2022. These estimates are based on archival maps and a selection of historical sources, which follows a hierarchy of criteria-based credibility and standardization. The information contained in MinCan covers 947 mines and provides information about their location (longitude and latitude in decimal), the company ownership, the principal commodities produced, and the years of operation (opening and closing dates). It is the first open access database to propose an exhaustive, free, and reliable compilation of the principal past and present mines producing in Canada. The geographic coordinates enable matching with other local, regional, and national databases, and allow for a wide range of research objectives to be met.

## Background & Summary

Renewable energy stimulates the global demand for critical minerals, which in turn revives public interest in the mining sector. Amid this rise in attention, more country-level and disaggregated databases are needed to research the complexities of social, economic, and environmental impacts of this industry. Available data on mineral activities are typically aggregated, which obscures the distinction between regional impacts and macro-level trends.

Addressing this timely need, this article introduces an open-source and quality-upgraded database to support research on mineral production. *MinCan*: *Past and Present Producing Mines of Canada, 1950–2022* database^1^ recounts Canadian mining between 1950 and 2022. This database holds significance for, among others, researchers from economics, geography, history, rural sociology, and environmental science. Moreover, sustainability assessments on the historical and recent impacts of the mining industries are possible using the location and dates of operations matched with environmental and socioeconomic databases^2^.

Mining in Canada presents several unique avenues for study. Together with Sweden and Chile, Canada has a long history of mining and a strong identity around the industry^3^. The industry has maintained significance since its historic boom during the Second World War^4^. Canadian mines cover vast territory and diverse commodities, creating possibilities for multiple regional cases as opposed to single mining-intensive regions. Moreover, although operational mines account for a small fraction of the national labor force^5^, mining regions in Canada are often mono-industrial where miners constitute a considerable share of labor. Finally, productivity and capitalization of the Canadian mining sector have been persistently high, centering around Toronto as a leader in global financial mineral markets. In the meanwhile, at the local level, profitability is characterized by booms-and-busts^6–8^.

The sample is constructed using historical maps issued by Natural Resources Canada^9^. In total, 947 mines are listed. For each mine, information is provided about its location (longitude and latitude in decimal), company ownership, principal commodities produced, and years of operation (opening and closing dates). For some mines, further information is documented including the name(s) of the mine, the size of the labor force, and relevant information about the mine’s history.

The approach taken for the *MinCan* database^1^ allows for a more accurate assessment of the impacts associated with mining activities at both regional and national levels, and for the use of various statistical techniques and database harmonization. The database’s open-access nature and geographical data allow for rich research and potential extensions.

## Database Contributions

The current gap in statistics results from the *uneven coverage in large databases* – the criteria for including a mine are not clearly defined and regions with extensive reporting are overrepresented. In addition, the *dispersion of information* among different sources calls for extensive harmonization of archival documentation. As a result, historical and abandoned mines are largely unrecorded and about 60% of the Canadian mines remain undocumented in the most common commercial database, *S&P Capital IQ Pro*^2^. I address the gap by providing the first long-run publicly available database for the Canadian mining industry. The *MinCan* database^1^ is novel for its mine-level information including the opening and closures dates and the geographic coordinates of sites.

The database has considerable advantages relative to existing sources (see general comparison in Table 1).

**Table 1:** Comparison of the databases.

	Historical Canadian Mine Database^10^	Mindat^12^	MinCan^1^
Number of Mines	14212 mines with duplicates	6534 mines	947 mines
Geographical coverage	uneven	**—**	even
Variables	50 variables	15 variables	33 variables
Variables’ availability	partial	partial	complete
Definition of Year of Operation	single opening and closure	not standardized (in text information)	multiple openings and closures
Period	1774–2019	approx. post-1850	1810–2022
Period coverage	partial coverage	partial coverage	full coverage after 1950
Sources	various sources (unspecified)	partially referenced (incl. non-expert)	various sources (following specific criteria)

*Historical Canadian Mines*^10,11^ hosted by the University of Saskatchewan proposes the largest sample size (14211 mines) and the longest historical coverage (1774 to today). Yet, the geographical coverage is uneven with an over-representation of the provinces of British Columbia and Ontario while Quebec is underreported. Also, the dates of operation are only accounted for in British Columbia and Ontario. Relative to it, I propose an exhaustive and reliable sample, an even survey of the whole Canadian geography and a continuous coverage of a specific period.

*Mindat* from The Hudson Institute of Mineralogy^12^ is a mineral database and mineralogical reference website with worldwide coverage. It is edited and created largely by volunteers. Relative to *Mindat*, credibility of data is enhanced. I prioritize provincial and territorial data over secondary sources like *Mindat* and non-experts in the collection of information.

Lastly, Natural Resources Canada hold additional mining statistics exist but are aggregated by the agency to protect confidentiality of respondents. For these data, the geographical location has not been assigned to each mine. The accessibility problem induced by confidentiality is a major limitation to mine-related research^2^. The present database offers location information for each mine and is available open source.

The *MinCan* database entails some shortcomings, and extensions would be pertinent. First, the presence of deposits is a core information to many geological analyses such as mineral deposit modelling estimates. The database would have further expanded its relevance to geological analysis by compiling information on the tonnages recovered and estimated reserves. In the same vein, it would be relevant to have indications of the scale of production associated with each mine. This could be attained with variables about the size of the labor force of each mine or the amount produced by the mine annually. Currently, protection of information constrains the availability of mine-level production data under confidentiality clauses. Further sources for accessing production and deposit data (tonnage and grade) include historical reports from government agencies, companies and SEDAR, as well as qualified technical reports utilizing standards such as NI 43-101 and JORC. Finally, the “company ownership.*i*” variables could be modified to indicate specific years of production associated with each company. The information was broadly available in the listed sources, but due to time constraints, this level of detail is not included in the database. It would allow analysis on variations in mine ownership and concentration across history.

## Methods

The mining industry is associated with a plethora of documentation including legal claims, business reports, documentation from governmental agencies, chronicles by non-specialist historians or accounts by municipalities about the local history. This wealth of information poses certain challenges for use in research, not least because it is dispersed and complex to harmonize. Some mines have greater availability of information than others due to factors such as administrative capacity of provinces, size of production, political importance, or the leadership of local citizens: this uneven spread creates potential ‘spotlight’ biases.

**Fig. 1:**
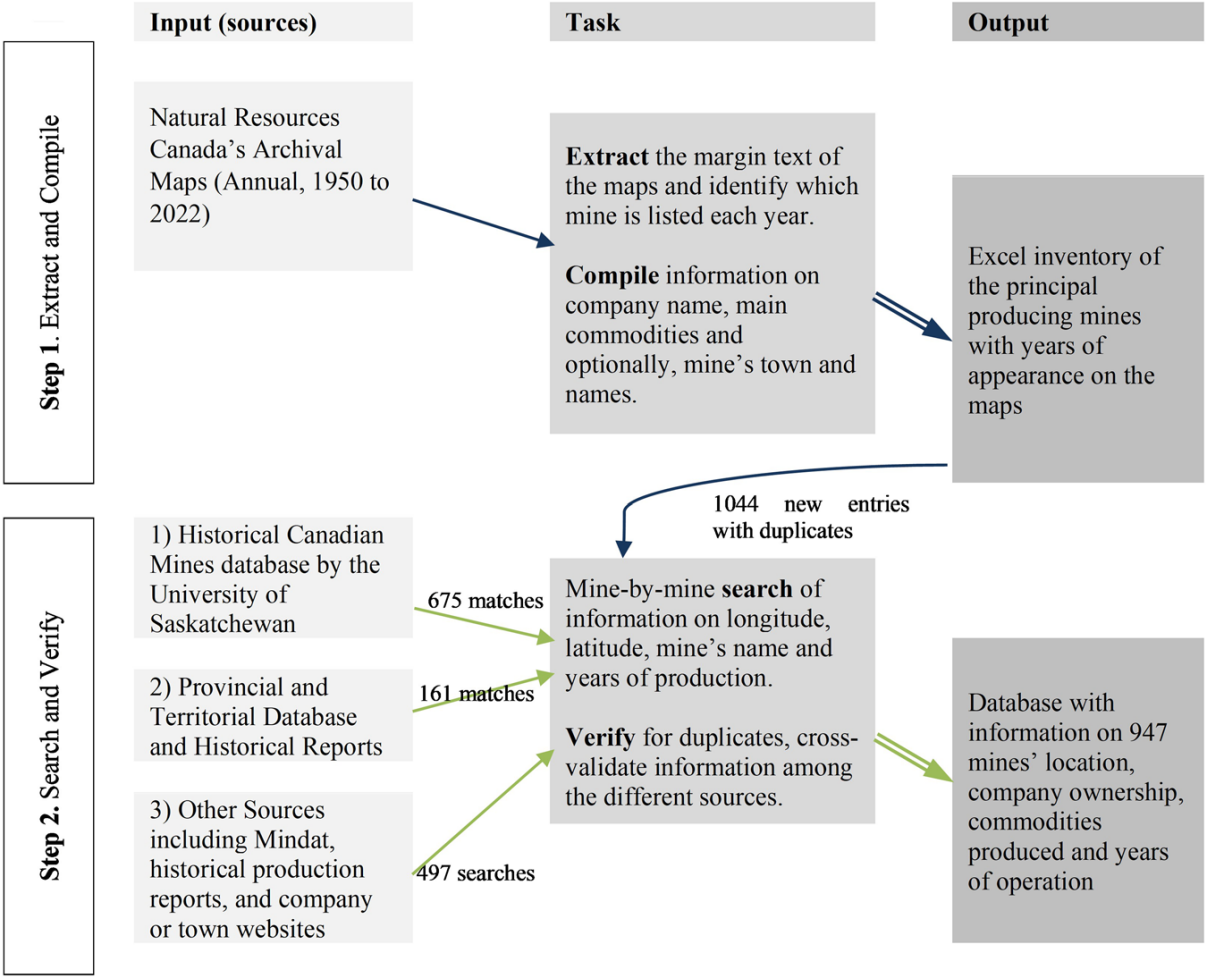
Schematic Representation of the Methodology.

To address these issues, the database results from a two-step procedure represented in Figure 1. The first step consists of the extraction and compilation of standardized mine entries. The resulting sample includes principal past and present mines in Canada with an explicit definition and an extensive coverage of the national territory. The second step is the search and verification for missing information based on a wider range of sources. Selection of these sources is based on a prioritization of sources’ credibility.

### Step 1. Extract and Compile

This first step is essential to construct a non-biased sample of the main mines. The selection directly follows what the national agency historically identified as the principal producing mines in the country. Indeed, the sample is constructed from Natural Resources Canada’s *Principal Mineral Areas, Producing Mines, and Oil and Gas Fields in Canada (Map 900 A)*^9^, first issued in 1947 and subsequently published annually since 1950.

Each map identifies the principal producing mines, counting approximately 200 mines for a given year. The maps list the name of the companies, the location and the principal commodities produced and sometimes, the name of the mine. This information was extracted for each year since 1950. The procedure resulted in the final sample of mines.

Principal producing mines included in the database are defined following Natural Resources Canada’s working definition: **“**A producing mine is a legitimate mining operation that has obtained all the required permits and is known to mine and ship at least one principal saleable mineral product. Excluded are operations that produce clay products and most construction materials (stone, sand and gravel, trap rock), placer mines, seasonal operations and mines operating under a permit intended for bulk sampling, test mining, small mine and/or exploration”^13^.

The focus on *principal* mines has three important implications. First, the level of precision of the database is enhanced. As the mines have a higher scale of production, they are generally easier to identify in historical sources and thus, cross-validation is facilitated. Second, the sample represents solely mines in operation, disregarding those that might be open but not producing. Third, the sample is focused on large producers. As small and large producers are affected differently by industrial changes and mineral price changes, no overgeneralization should be made.

### Step 2. Search and Verify

The second step consists of identifying the location of the mine, the years of production, and the different companies who operated the mine. This step allows identification and removal of duplicates, and triangulation of information. Three types of sources (source.i) are successively used until the missing information is gathered. For research pragmatic reasons, the collection of information prioritizes existing databases, all openly available. However, in the majority of cases (495/947), not all information is available, and a mine-specific investigation of secondary sources is necessary (source3). The use of the three sources is indicated at the mine-level in the database to increase transparency.

Firstly, the *Historical Canadian Mines*^10^ (source1 in the database) is led by Assistant Professor Donna Beneteau from the University of Saskatchewan. It is an estimable resource that contains geographical locations for 14211 mines from 1774 to 2019. The entire list of mines is reviewed one-by-one, as many cases necessitate a judgment to evaluate the best matching. Matching is validated based on when the production dates coincide. About half of the mines are matched with the *Historical Canadian Mines* database. Yet, uneven geographical coverage creates a strong bias. Significant amount of information is missing for the provinces of Saskatchewan and Quebec, possibly due to bias against French-language sources common in latter province.

Secondly, *Provincial and Territorial Sources* (source2 in the database) are collected by contacting the Geological Survey and/or the Department of Natural Resources and inquiring about the region-specific databases, references and inventories. After three waves of follow-up to office agents, responses allow to build a collection of sources for all provinces and territories, except Prince Edward Island, which has close to null mineral production (for Alberta^14^, British Columbia^15^, Manitoba^16^, New Brunswick^17^, Newfoundland-and-Labrador^18,19^, Northwest Territories^20,21^, Nova Scotia^22,23^, Nunavut^24,25^, Ontario^26^, Quebec^27^, Saskatchewan^28^ and Yukon^29,30^). Only a few provinces and territories have extensive information about average employees, mergers, history, and environmental claims.

Thirdly, *other sources* (source3 in the database) are consulted to remove duplicates and complete the database (497 mines were missing information). The *Abandoned Mines Information System* (AMIS) from Geology Ontario proposes information about known abandoned mine sites^31^; Quebec’s equivalent is the *List of Abandoned Mining Sites*^32^. Information about geographic coordinates and closure dates were extracted from both. *Mindat*^12^ is used for its extensive availability of geographic coordinates for mines. The information is sourced by the platform’s members in an open access manner, without guarantee scientific standards. Lastly, company reports, local history chronicles, amateur historical accounts, and media outlets are considered operation dates and the size of the labor force.

## Data Records

The resulting database is available in open access under a single Excel file from the Figshare platform^1^. The first sheet consists of the description and explanation of all variables (equivalent of Table 2) and the second sheet is the data itself.

**Table 2:** Description of the Variables.

Variable Label	Variable Name	Definition of the variable	Value Type	Optional
First Mining Company	company1	Name(s) of the mining companies that operated the mine. The list is non-exhaustive and includes consecutive owners and co-ownerships.	Textual	
Second Mining Company	company2			
Third Mining Company	company3			
Fourth Mining Company	company4			
Fifth Mining Company	company5			
Sixth Mining Company	company6			
Name of the Mine	name	Name(s) given to the mine at any point in time.	Textual	ü
Town of the Mine	town	Town where the mine is located.	Textual	ü
Province of the Mine	province	Province where the mine is located.	Textual	
Latitude of the Mine	latitude	Latitude coordinate of the mine. The format is decimal degree at the precision of 6 digits or more.	Numerical	
Longitude of the Mine	longitude	Longitude coordinate of the mine. The format is decimal degree at the precision of 6 digits or more.	Numerical	
Start of Production	open1	Years of production at the mine after 1949. close.i variables can indicate suspension or closure of operation: no distinction is made. In the identification of the years, production years were favored over years indicating the opening of closure of the mine. The variable takes the value of "open" when the mine is still in operation as of 2022. If the mine produced before 1949, it might be indicated under "information".	Date (YYYY)	
Suspension of Production	close1			
Reopening of Production	open2			
Second Suspension of Production	close2			
Re-Reopening of Production	open3			
Third Suspension of Production	close3			
All Commodities	commodityall	List of all the commodities produced by the mine.	Textual	
First Commodity	commodity1	Individually listed commodities produced by the mine. They are listed in order of priority. This is the same as the "commodityall" variable.	Textual	
Second Commodity	commodity2			
Third Commodity	commodity3			
Fourth Commodity	commodity4			
Fifth Commodity	commodity5			
Sixth Commodity	commodity6			
Seventh Commodity	commodity7			
Eight Commodity	commodity8			
Information about the Mine	information	Comments and information about the mine. Examples include the labor conflict/workplace accident, scale of production (small/large), whether it is a ghost town today and facts about the agency of indigenous groups.	Textual	ü
Canadian historical mines Source	source1	Indication (=1 if the source is used, =0 if the source is not used) that some information for this mine comes from the Historical Canadian Mines database [10].	Categorical (dummy)	
Provincial Sources	source2	Indication (=1 if the source is used, =0 if the source is not used) that some information for this mine is extracted from province- and territory-specific databases (Alberta^14^, British Columbia^15^, Manitoba^16^, New Brunswick^17^, Newfoundland-and-Labrador^18,19^, Northwest Territories^20,21^, Nova Scotia^22,23^, Nunavut^24,25^, Ontario^26^, Quebec^27^, Saskatchewan^28^ and Yukon^29,30^).	Categorical (dummy)	
Other Sources	source3	Indication (=1 if the source is used, =0 if the source is not used) that some information for this mine is extracted from the external weblinks provided (link1, link2 and link3).	Categorical (dummy)	
External web sources	link1	References for the secondary sources that have been consulted. It is often included tracking the sources of information and justify the decisions.	Website	ü
	link2			
	link3			

The core variables - available for each mine - concern the location of the mine, company ownership, commodities produced, and years of operation. For the optional variables, coverage includes the *Name of the mine* for 65% of the sample and *External web sources* (48% for which 27% with one reference, 16% with two and 7% with three). The *Information*, available for 30% of the database, includes the size of the labor force, special causes of closure, ecological events, information about the labor force, the municipality, and the participation of indigenous groups in the project or serves to catalogue metadata. Additional names for the mine or company are also reported.

### Commodities Produced by the Mines

Minerals produced are captured with multiple variables for *commodity.i*, with a maximum of eight commodities per mine. Approximately one hundred distinct minerals have been identified, with a substantial majority of them belonging to the category of metals. The main commodities are listed first. However, as some sources contradict each other and given that these may change in time, the numbering must be approached with caution. *First Commodity* with the highest frequency is gold; it is the main commodity for 248 mines (26% of the sample) followed by copper (13%), coal (10%), silver (5%), zinc, nickel, uranium, and iron.

### Geographical Distribution of the Mines

The location of the mine is communicated as a combination of two variables, *latitude* and *longitude*, expressed in decimal degrees with a minimum of six decimal places. This format facilitates the conversion to shape files and supports effective GIS analysis.

**Fig. 2:**
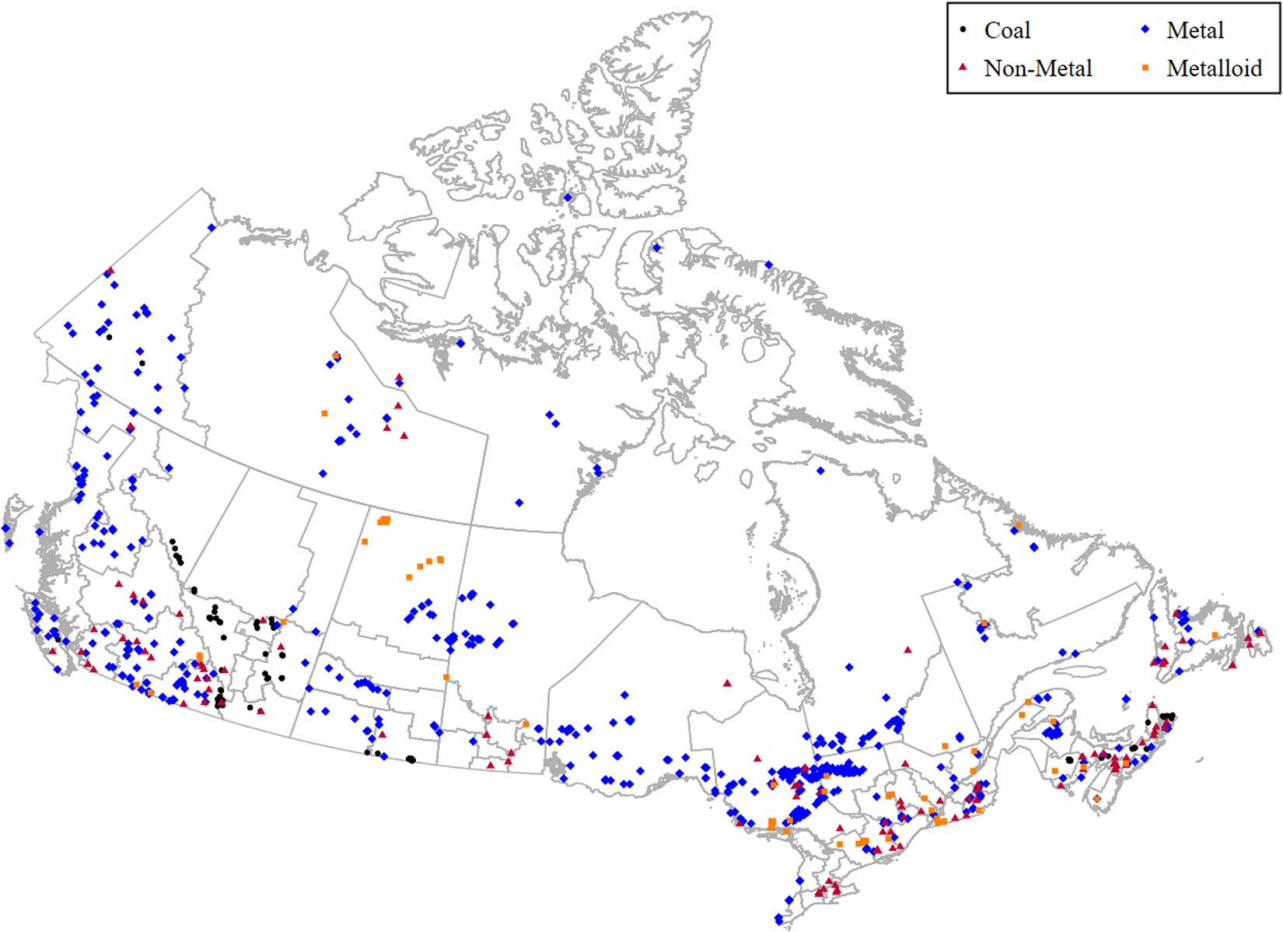
Spatial Distribution of Mines in the Sample per Mineral Type.

The variables indicate that the producing provinces are Ontario, Quebec, and British Columbia followed by Saskatchewan and Nova Scotia (see Figure 2). The historical mining region at the northern border of Quebec and Ontario has the highest concentration of mines. Considering that Nova Scotia has a relatively small territory, the presence of 55 mines in the province (5.81% of the sample for 0.55% of the area of Canada) also indicates a high density. The production of coal and uranium is concentrated in the province of Saskatchewan. In the latter decades, mines are also recorded in northern territories. New technologies enabled progressive exploration further north in Canada^4,33^.

### Company Ownership

Information about company ownership comes from various sources, but always includes the name listed in archival maps. In the rare cases where more than six companies are listed, the additional company names are reported under *information*. Company names are added when mine ownership changes or when a joint venture establishes dual ownership. Company ownership is associated with mines actively producing, excluding ownerships without operation. Some companies may not be reported and thus, the data should be used with caution.

Noting that a single mine can be operated by different companies over time, the most frequently listed companies are Teck (operating 23 mines throughout the period), HudBay Minerals Inc. (21 mines), Noranda Inc. (19 mines), Agnico Eagle Mines Ltd. (11 mines), Cominco Ltd. (11 mines), Domtar Ltd. (11 mines), and Falconbridge Ltd. (11 mines). Company ownership changes over time for 43% of the mines. The database indicates significant mergers and acquisitions in the late 20^th^ century. Teck Corporation Ltd., Rio Algom Ltd, Placer Dome Inc., Potash Corp of Saskatchewan, Noranda Inc., and Luscar Ltd. are associated with the reopening of operations or acquisition.

The database results from an effort to standardize the name of the companies toward common denominator as they might change over time or be listed under different names. External sources were consulted to ensure that these different names were associated with the same company.

### Years of Production

The variables associated with years of production are informative about the state of mineral production. The decline in mineral prices between 1996 and 2001 is shown in the database with the high number of closures relative to openings during the last decennia. Similarly, the increased demand for minerals during the post-war periods could be associated with the high number of openings during the 1950s and 1960s.

**Fig. 3:**
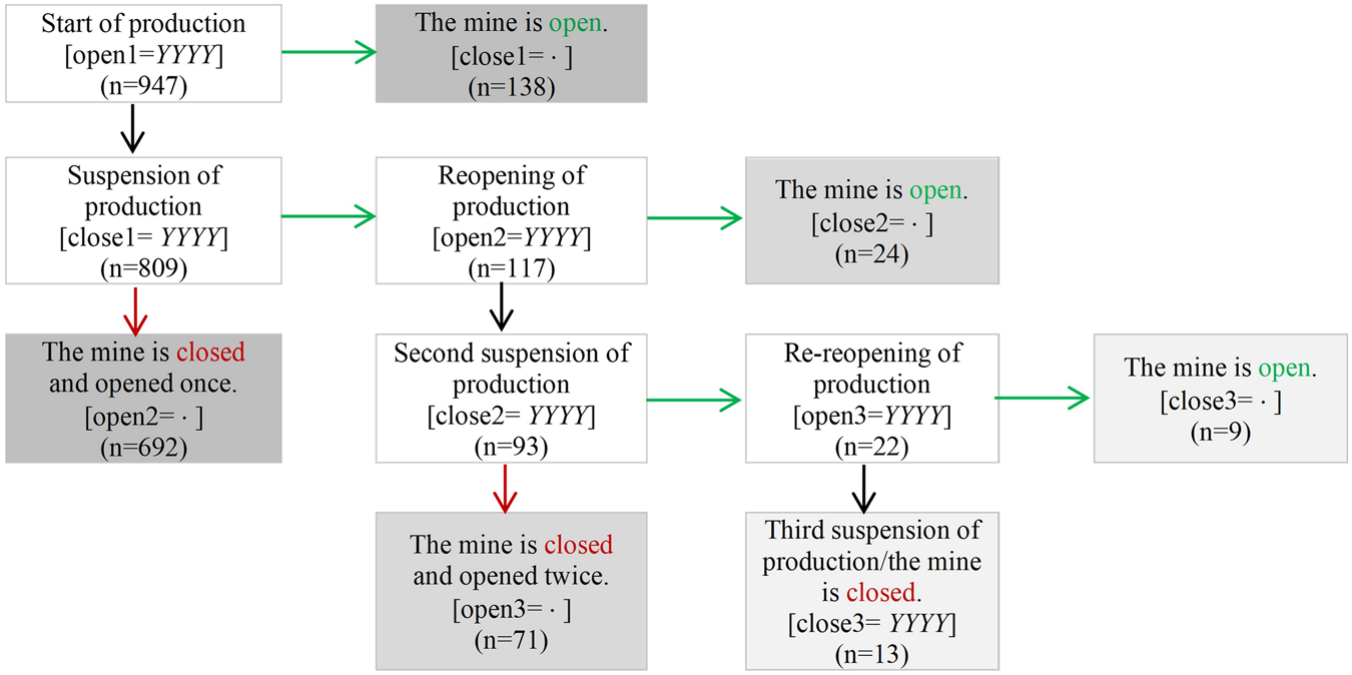
Flow Chart Representing the Changes in Status of Production.

As illustrated by Figure 3, The variables allow for multiple openings and closures to occur. The majority of the mines (73%) open and close only once. Meanwhile, 71 mines open and close twice, and 13 mines open and close three times. As of the end of 2022, 18% of the mines are open. With this information, the database can be used to investigate the length of time associated with the production of different minerals, the frequency of suspension of activities, and more generally different trends linked to fluctuations of industrial mining activities.

## Technical Validation

The validity of the sample rests on the conscious selection of a single source, the historical maps (900 A) from Natural Resources Canada. The national agency follows high quality control and relies on geologists’ expertise to survey the mines across all Canadian provinces and territories and all commodities. While additional mines were identified during the archival search, they have been excluded from the main database. Their inclusion would have biased the sample toward provinces and territories with more administrative capacity or mines with further documentation rather than those principal producers, which follow production trends.

The final sample was tested relative to production trends. Looking at the period between 1977 and 2022, Table 3 shows how the distribution of mines across different Canadian regions follows production trends. Discrepancies can be explained by the size of mines and profitability of different minerals, e.g., Nova Scotia’s salt production is associated with less commercial benefit than Saskatchewan’s coal and uranium production.

**Table 3:** Geographical Distribution of Mines and Production, 1997 to 2020.

Province or territory	MinCan^1^		Natural Resource Canada Production Statistics^34^	
	Number of Mines	Weight in the sample (%)	GDP for Mining and quarrying (million)	Weight in the sample (%)
British Columbia	63	15.07	4904	15.5
Alberta	23	5.50	1127	3.56
Saskatchewan	41	9.81	8238	26.04
Manitoba	17	4.07	913	2.89
Ontario	93	22.25	6609	20.89
Quebec	88	21.05	5485	17.34
New Brunswick	18	4.31	737	2.33
Prince-Edward-Island	0	0.00	3	0.01
Nova Scotia	22	5.26	189	0.6
Newfoundland and Labrador	25	5.98	1916	6.06
Yukon	11	2.63	93	0.3
Northwest Territories	8	1.91	1079	3.41
Nunavut	9	2.15	339	1.07
Total - Canada	418	100	31632	100

In addition to checks for duplicates, cross-validation has been carried out throughout the two-step procedure. During Step 1, two verifications were conducted. First, to validate the identification of the sample, the number of mines every year was compared in the database^1^ and historical maps^9^. In case of discrepancies, the database was revised. Second, to identify the presence of duplicates, further verifications are made when the company name listed is different or another entry has a similar location and commodities. This verification allows distinguishing between a mine closure and a change in ownership. To verify the accuracy of Step 2, an external researcher conducted the same task with a sub-sample to validate the conclusions and estimate the margin of error. The results of the matching are the same, confirming the validity of the data collection.

Lastly, the geographic location accuracy has been tested on a subsample of 40 mines using the geological survey from the Ontario Mining Inventory^26^. The inventory contains precise official information on the location of mineral operations for this specific province. The comparison exhibits close to equal matching with variations occurring only at the 1/10000 decimal degree (11.13 meters).

## Data Availability

The author reports there are no custom code used.
